# Diabetic Retinopathy Screening Adherence

**DOI:** 10.22336/rjo.2024.76

**Published:** 2024

**Authors:** Pasha Anvari, Reza Mirshahi, Faezeh Hashemi Nezhad, Alireza Helal Birjandi, Kimia Daneshvar, Ramin Fakhar, Atefeh Ghomashi, Khalil Ghasemi Falavarjani

**Affiliations:** 1 Eye Research Center, The Five Senses Health Institute, Rassoul Akram Hospital, Iran; University of Medical Sciences, Tehran, Iran; 2 Department of Ophthalmology, School of Medicine, Iran University of Medical Sciences, Tehran, Iran; 3 School of Medicine, Tehran University of Medical Sciences, Tehran, Iran; 4 Stem Cell and Regenerative Medicine Research Center, Iran University of Medical Sciences, Tehran, Iran

**Keywords:** diabetic retinopathy, screening guidelines, COVID, adherence, AAO = The American Academy of Ophthalmology, ADA = the American Diabetes Association, anti-VEGF = anti-vascular endothelial growth factor therapy, BCVA = best-corrected visual acuity, COVID = Coronavirus disease, DM = diabetes mellitus, DR = Diabetic retinopathy

## Abstract

**Objective:**

To evaluate the adherence rate of diabetic subjects to the retinopathy screening program and to identify the characteristics associated with non-compliance to regular screening.

**Methods:**

A cross-sectional study involving 240 patients with diabetes who attended outpatient non-ophthalmology clinics at four tertiary university hospitals between March 2020 and March 2021 was conducted. A validated questionnaire collected data that included socio-demographic variables, characteristics of diabetes, diabetes-related complications and comorbidities, knowledge and attitudes toward DR and its screening, and barriers to DR screening. Univariate and multivariate logistic regression was employed to identify the factors associated with adherence to the annual diabetic eye examination. Adherence was defined as a history of the dilated ophthalmic exam in the past year and subsequent follow-ups recommended by the ophthalmologist.

**Results:**

The participants had an average age of 59 ± 14.2 years, and 53% were females. The average duration of diabetes was 108 ± 89.62 months. Based on the last ophthalmic examination, 50.8% of patients were non-adherent to the suggested DR screening guidelines. A history of smoking (p-value=0.013, 95%CI for OR: 1.21-5.10), lower education levels (p-value=0.045, 95%CI for OR: 1.02-3.82), and not clarifying the necessity of ophthalmic examination by primary physicians (p-value < 0.001, 95%CI for OR: 2.19-12.35) were significantly associated with non-adherence. Among non-adherent subjects, 38% reported fear of the COVID-19 pandemic, 21.3% cited the lack of information regarding DR screening, 10.7% cited lack of access to the ophthalmologist, 5.7% cited financial problems, and 10.7% noted a lack of support from family and friends as the main reason for non-attendance to the annual eye care.

**Discussions:**

This study revealed a non-compliance rate with DR screening guidelines that surpasses pre-pandemic figures. Although younger participants demonstrated a higher likelihood of recent eye care, this correlation lost significance in multivariate analysis, potentially reflecting education and technology utilization disparities. Despite uniform insurance coverage, socioeconomic factors, such as transportation challenges, may impede adherence. Furthermore, individuals informed by healthcare providers about the necessity of eye exams exhibited greater compliance; however, many newly diagnosed patients remained unaware of the risks associated with DR. The COVID-19 pandemic emerged as a prominent barrier, with numerous patients citing it as a reason for their non-compliance due to disruptions in clinical care.

**Conclusions:**

Half of the diabetic subjects attending tertiary hospitals for non-ophthalmic complaints were non-adherent to recommended retinopathy screening. Lower education, smoking, and unawareness of the necessity of annual eye care had the strongest association with non-compliance. Fear of contracting COVID-19 was the most prevalent barrier to the eye examination. These findings underscore the necessity for targeted patient education initiatives, particularly among those with lower education levels and smokers.

## Introduction

Diabetic retinopathy (DR), a microvascular complication of diabetes mellitus (DM), is one of the foremost causes of preventable vision loss in adults aged 20-74 years worldwide [1,2]. The prevalence of DR is increasing with the projected increase in diabetic individuals [3,4]. There are various strategies for managing DR, including primary prevention to delay the onset of DR in diabetic patients, secondary prevention to halt the progression of DR, and tertiary prevention using laser photocoagulation, intravitreal anti-vascular endothelial growth factor therapy (anti-VEGF) injections, and vitreoretinal surgery to treat complications [5].

Detecting DR in the initial stages can mitigate the vision-related functional impact of diabetes [6]. Timely screening can control up to 90% of diabetes-related vision [7]. The American Academy of Ophthalmology (AAO) and the American Diabetes Association (ADA) recommend dilated eye screening for DM at least annually. However, the ADA states that in the absence of DR signs in one or more ophthalmic examinations and with adequate glycemic control, the screening interval can be extended to every two years [8,9]. Nevertheless, adherence to the DR screening examination ranges from 23% to 65% in the US [10,11]. Several barriers have been reported to be associated with the poor adherence of the patients to routine eye examinations, including younger age, lower income, lower education, race, insulin use, fewer comorbidities, less diabetes education, lower adherence to the oral hypoglycemic agents, hemoglobin A1C >9%, cost, lack of insurance, no timely diabetic foot examination, no convenient access to the nearest ophthalmologist, and living in the rural areas [10,12-17].

The COVID-19 pandemic has adversely affected routine screening for diseases. For instance, the monthly screening rates for the three most prevalent cancers in the US experienced a steep drop during the COVID-19 pandemic, leading to a screening deficit of 9.4 million individuals [12]. Previous studies have revealed a 30 to 100% reduction in the rate of intravitreal injections of anti-VEGF with consequent visual loss in diabetic individuals during the COVID-19 pandemic [13]. Identifying the factors contributing to non-compliance with screening protocols could assist health policymakers in devising strategies to alleviate these factors and potentially diminish the ensuing visual impairment and burden.

This study examined adherence to the recommended ophthalmic examination among diabetic individuals attending outpatient clinics for non-ophthalmic complaints. It explored the predictors of non-adherence and barriers to the eye care guidelines in the COVID era.

## Methods

This cross-sectional study enrolled subjects with type 2 DM who presented to the outpatient non-ophthalmology clinics of four tertiary university hospitals (Rassoul Akram, Firouzgar, Haft-e-Tir, and Firouzabadi) between March 2020 and March 2021. The diagnosis of DM was based on self-report and/or medical records. The study protocol (registration number IR.IUMS.REC.1400.352) received approval from the Iran University Ethics Committee and complied with the Declaration of Helsinki. Participants provided written informed consent.

Subjects were classified as adherent or non-adherent based on the last ophthalmic examinations. Adherent patients had their ophthalmic examination within the past year and followed the ophthalmologist’s recommended follow-up schedule based on dilated fundus examination [8].

A survey explored the associated factors of non-adherence to DR screening guidelines by examining various attributes, including age, sex, duration of diabetes, history of diabetic retinopathy or other microvascular diabetic complications (such as nephropathy or neuropathy), history of hypertension, history of cerebrovascular disease, history of cardiovascular disease, history of diabetic foot, history of other endocrinopathies (such as thyroid disease), health insurance coverage, a current habit of smoking regularly, educational status (college or higher vs. lower education), recent hospitalization (within the last 12 months for any reason), residence (urban vs. rural), living alone vs. having a supportive family, type of diabetes treatment (oral agents only vs. insulin ± oral agents) and knowledge of the need for annual screening ophthalmic examinations. The content validity of the survey was assessed using experts’ opinions. In addition, an inquiry was conducted in the cohort of non-adherent subjects to identify the underlying reasons for non-adherence.

Two trained investigators (FHN, AHB) interviewed them and completed data collection forms. When available, medical records were also reviewed.

## Results

Two-hundred-forty patients (47% male) with a mean age of 59.07 ± 14.26 years (range: 24-90 years) and a mean duration of 108.07 ± 89.62 months (range: 1-480 mo.) of type 2 diabetes were included. The data were gathered from patients attending 21 non-ophthalmology wards. Based on the last ophthalmic examination, 122 patients (50.8%) were non-compliant with ophthalmic exam guidelines.

**Table 1** presents the details of the univariate analysis. The non-adherent group had significantly higher age, smoking status, urban residence, and prevalence of other endocrinopathies and cardiovascular diseases than the adherent group. Moreover, their primary care physicians or endocrinologists provided less information about the screening guidelines to the non-adherent group.

**Table 1:** Univariate analysis of factors linked to non-adherence to the diabetic retinopathy screening protocol

	Non-Adherent (n=122)	Adherent (n=118)	P-value^*^
Age (mean ± SD)	62.45 ± 14.21	55.57 ± 13.50	<0.001
Gender (% Female)	51.6%	55.9%	0.505
Duration of DM (mean ± SD months)	117.89 ± 95.0	97.91 ± 82.87	0.088
Residential location (% urban)	60.7%	74.6%	0.022
Recent hospitalization (% yes)	26.2%	28.8%	0.654
Current smoker (% yes)	27.9%	14.4%	0.012
The importance of regular Ophthalmic exams is clarified by the primary care physician (% yes)	68.9%	93.2%	<0.001
Education (% College or higher levels)	45.9%	71.2%	<0.001
Insurance covering the DR screening (% yes)	82.8%	85.6%	0.969
Mode of diabetes treatment (% oral agents only)	38.5%	39%	0.402
Living companion (% living alone)	14.8%	8.5%	0.134
History of Retinopathy (% yes)	20.5%	16.1%	0.381
History of Neuropathy (% yes)	39.3%	28.8%	0.086
History of Nephropathy (% yes)	40.2%	38.1%	0.748
History of Diabetic Foot (% yes)	18.9%	11%	0.093
History of Cardiovascular disease (% yes)	49.2%	28.8%	0.001
History of HTN (% yes)	56.6%	54.2%	0.718
History of other Endocrinopathies (% yes)	5.7%	13.6%	0.045
History of Cerebrovascular Diseases (% yes)	8.2%	3.4%	0.123
Overall Diabetic Complications (% yes)	57.4%	52.5%	0.452
Overall Presence of Comorbidities (% yes)	83.6%	80.5%	0.532

* Calculated by binary logistic regression

In multivariate logistic regression analysis, the predictors of non-adherence were a history of smoking (p-value = 0.013, OR=2.48 95% CI for OR: 1.21-5.10), lower level of education (high school and lower vs. university education, p-value = 0.045, OR=1.97 95% C. for OR: 1.02-3.82) and lack of knowledge of the necessity of screening ophthalmic examination (p-value < 0.001, OR=5.19 95% CI for OR: 2.19-12.35) (**Table 2**).

**Table 2:** Multivariate Analysis

Variable	B	P-value
Age	-0.006	0.610
Residential location	0.435	0.194
Duration of DM	-0.001	0.194
History of Neuropathy	0.074	0.834
History of Diabetic Foot	0.316	0.478
History of Cardiovascular Disease	0.505	0.120
History of other Endocrinopathies	-0.975	0.086
Current Smoker	0.909	0.013
Education	-0.678	0.045
The primary care physician has clarified the importance of regular ophthalmic exams	-1.647	<0.001

Fear of COVID-19 transmission (38%), lack of information regarding DR screening (21.3%), lack of access to an ophthalmologist (10.7%), financial problems (5.7%) and lack of support from friends or family members (10.7%), were the main reasons for non-adherence among non-adherent subjects. Notably, fifty-one patients (41.8%) reported no specific reason for non-adherence.

## Discussion

Our study demonstrated that 50.8% of participants did not comply with the DR screening guidelines, a slightly higher non-adherence rate than in previous studies before the COVID-19 pandemic (23.5% to 48.8%) [15-17].

We identified factors associated with non-adherence to diabetic eye care, including lower education levels, smoking, and insufficient knowledge about the value of eye examination by primary physicians. In univariate analysis, younger subjects were more likely to have eye care within the last year, but this association was insignificant in multivariate analysis. This difference may indicate that younger subjects were more educated and used modern information technologies more. This finding differed from previous studies and could reflect cultural discrepancies [10,16].

Previous reports have linked patients’ socioeconomic status to poor adherence and higher retinopathy progression rates among less educated individuals [10,16,18]. Our results corroborated these findings. However, both groups in our study had equal insurance coverage. Thus, other factors affecting low socioeconomic groups, such as transportation difficulties, might influence the outcome.

In this study, adherent individuals were more likely to have received information from their physicians regarding the necessity of ophthalmic examination and potential complications of diabetes, such as vision loss. However, many newly diagnosed individuals were unaware of the importance of regular eye examinations and the risk of DR, which could affect 6% of them [19]. Previous exposure to DR complications among friends and relatives motivates adherent individuals to visit an ophthalmologist regularly, as they fear losing their vision [20]. However, this fear may also have a paradoxical effect and deter them from participating in screening [21].

Paksin-Hall et al. reported that individuals who attended diabetic management classes were more inclined to undergo a diabetic eye examination within the past year [16]. Hartnett et al. found that half of the patients were unaware of the need for an annual ophthalmic examination [22]. Lack of knowledge of the rationale behind the yearly retinal examination was a significant barrier for many patients [23,24].

Murchison et al. showed that individuals who smoked exhibited lower compliance with timely retinal examinations than those who did not smoke, with a reduced odds ratio of 0.72 [25]. Gibson et al. corroborated that smoking conferred a greater risk of non-adherence of more than 1.3 times [19]. Despite the link between smoking and macular degeneration, the independent effect of smoking on adherence to eye care stemmed from the simultaneous presence of other symptomatic health conditions in smokers, which compelled them to prioritize acute medical conditions over asymptomatic issues and screening tests [20,26]. Moreover, the lack of information regarding the necessity of routine retinal screening among smokers due to their lower-level education can also account for the higher adherence of non-smokers [21].

In our study, 38% of patients attributed non-adherence to the COVID-19 pandemic. COVID-19 profoundly affected clinical care and necessitated a transition to telemedicine in clinical practices. However, telemedicine has drawbacks compared to direct examinations by a physician, especially an ophthalmologist. The emergence of non-mydriatic cameras and artificial intelligence platforms for image analysis has opened a new window in identifying high-risk patients through pre-screening [1]. The COVID-19 pandemic disrupted ophthalmic care provision, including regular follow-up visits and anti-VEGF injections, dramatically affecting patients’ best-corrected visual acuity (BCVA). A study from the Department of Ophthalmology at China Medical University First Hospital found that the mean duration of treatment interruption was 5.3 ± 0.8 weeks, which led to a significant deterioration in BCVA at the subsequent visits [22]. Similarly, a remarkable decline in BCVA was observed in all the patients who required intravitreal injections of anti-VEGF during the COVID-19 lockdown at the Jordan University of Science and Technology, where the mean delay was 6.2 ± 1.4 weeks [24].

Our study had several limitations that should be acknowledged. First, we excluded patients with type-1 DM who might have faced different challenges to adherence. Second, our results were restricted to subjects attending outpatient clinics. Thus, our findings might not have been generalizable to the general population. In addition, due to the limited sample size, we did not conduct subgroup analysis for different clinics.

## Conclusion

In conclusion, we found a high non-adherence rate to DR screening guidelines during the COVID-19 pandemic. We highlighted the need for patient education on the importance of regular ophthalmology check-ups, especially among smokers and those with lower educational attainment. This study might provide valuable insights for health policymakers to reduce the risk of preventable blindness from DR.

## Data Availability

The datasets used and analyzed during the current study are available from the corresponding author upon reasonable request.
